# Coffee and Caffeine Ingestion Have Little Effect on Repeated Sprint Cycling in Relatively Untrained Males

**DOI:** 10.3390/sports4030045

**Published:** 2016-08-29

**Authors:** Neil Clarke, Harry Baxter, Emmanuel Fajemilua, Victoria Jones, Samuel Oxford, Darren Richardson, Charlotte Wyatt, Peter Mundy

**Affiliations:** School of Life Sciences, Coventry University, Coventry CV1 5FB, UK; baxterh@uni.coventry.ac.uk (H.B.); fajemile@uni.coventry.ac.uk (E.F.); jonesv10@uni.coventry.ac.uk (V.J.); apx327@coventry.ac.uk (S.O.); richa190@uni.coventry.ac.uk (D.R.); wyattc2@uni.coventry.ac.uk (C.W.); ab9674@coventry.ac.uk (P.M.)

**Keywords:** caffeine, coffee, repeated sprints, cycling

## Abstract

The present study investigated the effect of ingesting caffeine-dose-matched anhydrous caffeine or coffee on the performance of repeated sprints. Twelve recreationally active males (mean ± SD age: 22 ± 2 years, height: 1.78 ± 0.07 m, body mass: 81 ± 16 kg) completed eighteen 4 s sprints with 116 s recovery on a cycle ergometer on four separate occasions in a double-blind, randomised, counterbalanced crossover design. Participants ingested either 3 mg·kg^−1^ of caffeine (CAF), 0.09 g·kg^−1^ coffee, which provided 3 mg·kg^−1^ of caffeine (COF), a taste-matched placebo beverage (PLA), or a control condition (CON) 45 min prior to commencing the exercise protocol. Peak and mean power output and rating of perceived exertion (RPE) were recorded for each sprint. There were no significant differences in peak power output (CAF: 949 ± 199 W, COF: 949 ± 174 W, PLA: 971 ± 149 W and CON: 975 ± 170 W; *p* = 0.872; $\eta_{P}^{2}$ = 0.02) or mean power output (CAF: 873 ± 172 W, COF: 862 ± 44 W, PLA: 887 ± 119 W and CON: 892 ± 143 W; *p* = 0.819; $\eta_{P}^{2}$ = 0.03) between experimental conditions. Mean RPE was similar for all trials (CAF: 11 ± 2, COF: 11 ± 2, PLA: 11 ± 2 and CON: 11 ± 2; *p* = 0.927; $\eta_{P}^{2}$ = 0.01). Neither the ingestion of COF or CAF improved repeated sprint cycling performance in relatively untrained males.

## 1. Introduction

The majority of research into caffeine as an ergogenic aid has focused on endurance performance, although there is also evidence to suggest that caffeine positively affects short-term high-intensity performance [1]. A number of studies report significant improvements in short-term high-intensity [2,3,4] and repeated-sprint performance [5,6,7] following the ingestion of between 5 and 9 mg·kg^−1^ of caffeine. However, Paton et al. [8] (6 mg·kg^−1^ of caffeine) and Glaister et al. [9] (2–10 mg·kg^−1^ of caffeine) reported that caffeine had no effect on repeated sprint performance.

The leading hypothesis as to how caffeine could have an ergogenic effect during anaerobic exercise is via the facilitation of central effects by antagonising adenosine receptors, thereby inhibiting the negative effects of adenosine on neurotransmission, arousal and pain perception [10]. The ingestion of caffeine also stimulates the central nervous system which increases alertness and focus [11]. A number of other possible mechanisms have been attributed to the ergogenic effects of caffeine including central nervous system facilitation [12] and mobilisation of intracellular calcium [13], as Tarnopolsky and Cupido [14] suggested that caffeine increases calcium release from the sarcoplasmic reticulum. Furthermore, Tarnopolsky [15] suggested that caffeine increases motor-unit recruitment, contractility of skeletal muscle and force production by enhanced neuromuscular transmission, and therefore maximal muscle activation. There is also evidence to suggest that caffeine may enrich the sodium-potassium pump within skeletal muscle and enhance excitation contraction coupling [10], which aids improvements in physical performance [16]. However, Astorino and Roberson [11] concluded that the mechanism by which caffeine provides an ergogenic effect in short-term, high intensity exercise is likely to be multifactorial. Central factors such as adenosine antagonism may be the most probable mechanism, with alterations in perceived exertion, reaction time, cognition, and mood also having an influence on performance.

The predominant dietary source of caffeine is coffee [17], although there is debate about the effectiveness of coffee as a performance enhancer. Costill, Dalsky and Fink [18] and Wiles et al. [19] concluded that coffee consumption enhances high-intensity exercise performance. However, Graham et al. [20] suggested that only anhydrous caffeine improved exercise performance when running at 85% VO_2max_ and proposed that chlorogenic acids, and possibly other ingredients within the coffee, nullified the ergogenic benefits of caffeine. More recently, Hodgson, Randell and Jeukendrup [21] reported that during a cycling time trial, dose matched coffee and caffeine ingestion were beneficial to exercise performance by similar magnitudes. Furthermore, Trexler et al. [22] concluded that coffee is a suitable source of caffeine to improve repeated sprint performance. The inconsistency in performance outcomes of coffee between studies may be due to brands of coffee used as there has been shown to be significant variances in chlorogenic acid content [23], as well as exercise modality [24], caffeine solution strengths within the coffee, and participant training status [25]. Furthermore, the majority of the aforementioned investigations focused on endurance-based protocols. In contrast, Wiles et al. [19] observed that 1500 m running performance improved significantly following the ingestion of coffee. Therefore, both the coffee and caffeine have the potential to improve repeated sprint performance during cycling. Consequently, the aim of the present study was to determine the effect of ingesting caffeine-dose-matched anhydrous caffeine or coffee on the performance of repeated sprints on a cycle ergometer.

## 2. Materials and Methods

### 2.1. Study Design

Participants completed eighteen 4 s sprints separated by 116 s recovery on a cycle ergometer (WattBike Pro, WattBike Ltd., Nottingham, UK) on four separate occasions in a double-blind, randomised, counterbalanced crossover design following the ingestion of caffeine, caffeinated coffee, a placebo beverage or a control condition. The exercise protocol was designed to replicate team-sport-based exercise [26]. The study was approved by the local Ethics Committee and undertaken in accordance with the Declaration of Helsinki.

### 2.2. Participants

Twelve recreationally active males (mean ± SD age: 22 ± 2 years, height: 1.78 ± 0.07 m, body mass: 81 ± 16 kg) were made fully aware of the exact procedures, including any risks and benefits of participation in the study before providing written informed consent.

### 2.3. Experimental Trial

Prior to the first session, a familiarisation session was conducted where the testing protocol and equipment was explained and practiced. Participants arrived at the laboratory at least 3 h postprandial having abstained from caffeine, alcohol, and strenuous activity for 12 h, and recorded their dietary intake for 48 h before the initial experimental trial. This diet was then replicated before the subsequent experimental trials. Participants consumed the experimental beverages 45 min prior to exercise, in agreement with previous studies [27]. Participants ingested either 3 mg·kg^−1^ of caffeine (MyProtein; Manchester, UK; CAF), 0.09 g·kg^−1^ coffee (Nescafé Original, Nestlé, Gatwick, UK), which contained 3 mg·kg^−1^ of caffeine (COF), a taste-matched placebo beverage (Whole Earth Organic coffee alternative, Wessanen, Surrey, UK) with no caffeine content (0 mg·kg^−1^; PLA), or a control condition where no beverage was ingested (CON) 45 min prior to commencing the exercise protocol. Nescafé original coffee was used as Hodgson, et al. [21] reported that it contains 3.4 g of caffeine per 100 g of coffee, meaning that each participant consumed 0.09 g·kg^−1^ of coffee to achieve the 3 mg·kg^−1^ of caffeine required. All substances were dissolved in 300 mL of hot water (62.4 ± 1.4 °C). A dose of 3 mg·kg^−1^ of caffeine has previously been demonstrated to provide an ergogenic effect [28].

Participants completed a repeated sprint test, based on the protocol described by Zimmermann and Landers [29] on a cycle ergometer. Participants adjusted the saddle and handle bars for comfort and these adjustments remained consistent for all trials. Participants performed a two-minute warm up at 70–80 rpm with air resistance of “level 1” immediately followed by eighteen 4 s maximal sprints at air brake resistance level 3, magnet setting 1 [30]. Each 4-s sprint was followed by 96 s of active recovery (continuous cycling at 70 rpm) and then 20 s of passive recovery (rest). Peak and mean power output and rating of perceived exertion (RPE; Borg [31]) were recorded for each sprint. A capillary blood sample was then drawn from the index finger for determination of blood lactate concentration (*Biosen* HbA1c, EKF-diagnostic GmbH, Magdeburg, Germany) immediately prior to the start (post-warm-up) and at the completion of each trial in order to assess physiological stress. Participants received no feedback about their performance until all three trials were completed; this was to reduce bias, as was the procedure in previous studies [21].

### 2.4. Statistical Analysis

Data are reported as the mean ± the standard deviation (SD). In addition, as the responses to low doses of caffeine are often variable and athletes need to determine whether the ingestion of caffeine is ergogenic on an individual basis [28], the mean individual performances for each condition are also presented. The Shapiro-Wilk test was applied to the data in order to assess for a normal distribution. All variables were analysed with a two-way ANOVA with repeated measures, with the exception of overall peak and mean power output, which were assessed using a one-way ANOVA with repeated measures. Sphericity was analysed by Mauchly’s test of sphericity followed by the Greenhouse-Geisser adjustment where required. Where any differences were identified, pairwise comparisons with Bonferroni correction were used in order to show where they lay. All statistical procedures were conducted using IBM SPSS Statistics for Windows, Version 22.0 (IBM Corp., Armonk, NY, USA). An alpha level of *p* < 0.05 was considered statistically significant and interpretations were made in accordance with the suggestions by Curran-Everett and Benos [32]. Furthermore, effect sizes using partial eta squared ($\eta_{P}^{2}$) were calculated, and were defined as trivial, small, moderate or large [33].

## 3. Results

**Figure 1:**
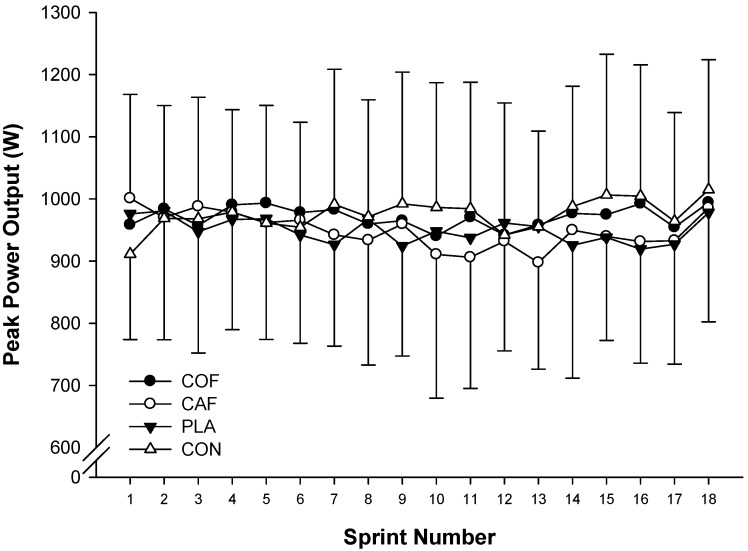
Peak power output during repeated cycling sprints following the ingestion of caffeinated coffee, caffeine, placebo and a control condition.

**Figure 2:**
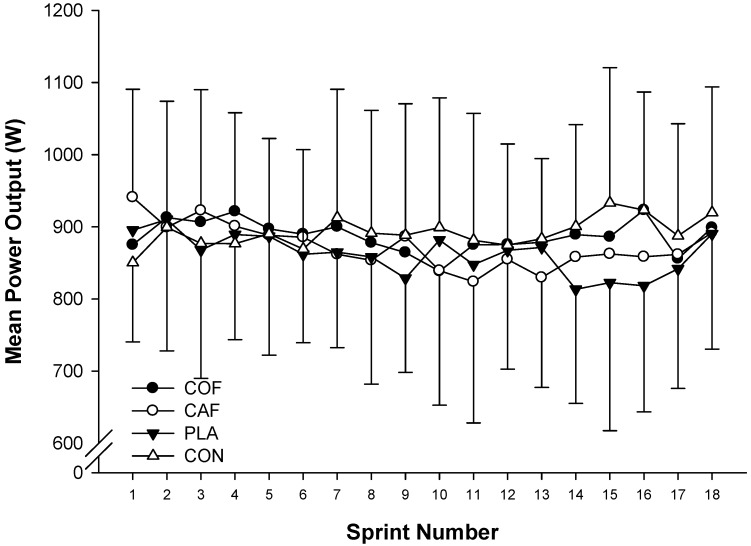
Mean power output during repeated cycling sprints following the ingestion of caffeinated coffee, caffeine, placebo and a control condition.

**Figure 3:**
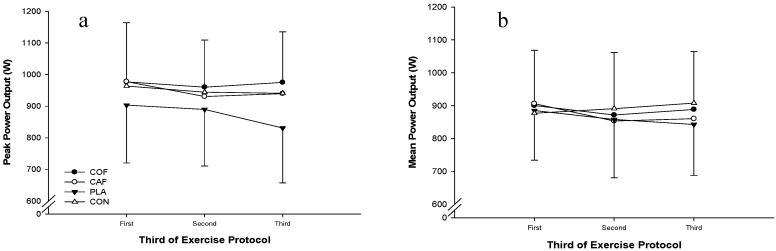
Mean peak (**a**) and mean (**b**) power output during each third (i.e., six sprints) of the exercise protocol following the ingestion of caffeinated coffee, caffeine, placebo and a control condition.

**Figure 4:**
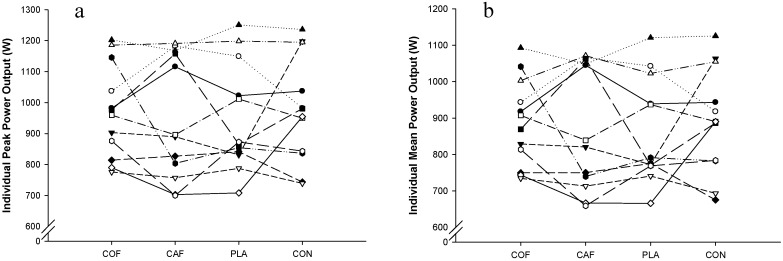
Mean individual peak (**a**) and mean (**b**) power output during repeated cycling sprints following the ingestion of caffeinated coffee, caffeine, placebo and a control condition.

There were no significant differences in peak power output (F_3,33_ = 0.233; *p* = 0.872; $\eta_{P}^{2}$ = 0.03; Figure 1) or mean power output (F_3,33_ = 0.308; *p* = 0.819; $\eta_{P}^{2}$ = 0.03; Figure 2) between experimental conditions. In addition, there was a trend for peak power output (F_17,187_ = 1.582; *p* = 0.072; $\eta_{P}^{2}$ = 0.13) and mean power output (F_5,51_ = 2.155; *p* = 0.078; $\eta_{P}^{2}$ = 0.16) to decline throughout the protocol. However, there were no significant differences in the fatigue index (COF: 5%, CAF: −2%, PLA: 2%, CON: 12%; F_3,33_ = 1.391; *p* = 0.263; $\eta_{P}^{2}$ = 0.11) and peak power observed during the first sprint (COF: 958 ± 179 W, CAF: 1001 ± 167W, PLA: 976 ± 224 W, CON: 911 ± 137 W; F_3,33_ = 1.278; *p* = 0.298; $\eta_{P}^{2}$ = 0.10) during each condition. Analysis of the peak power output (F_3,33_ = 0.232; *p* = 0.873; $\eta_{P}^{2}$ = 0.02; Figure 3a) and mean power output (F_3,33_ = 0.307; *p* = 0.820; $\eta_{P}^{2}$ = 0.03; Figure 3b) for each third of the exercise protocol revealed no significant differences between conditions, and there was no main effect of time with regards to peak power output (F_2,22_ = 1.180; *p* = 0.326; $\eta_{P}^{2}$ = 0.10), although mean power output was greater in the first third of the protocol compared with the second (F_2,22_ = 4.694; *p* = 0.020; $\eta_{P}^{2}$ = 0.30). Furthermore, there were no significant differences in overall peak power output (COF: 949 ± 174 W, CAF: 949 ± 199 W, PLA: 971 ± 149 W, CON: 975 ± 170 W; F_3,33_ = 0.233; *p* = 0.872; $\eta_{P}^{2}$ = 0.02) or mean power output (COF: 862 ± 44 W, CAF: 873 ± 172 W, PLA: 887 ± 119 W, CON: 892 ± 143 W; F_3,33_ = 0.308; *p* = 0.819; $\eta_{P}^{2}$ = 0.03) between experimental conditions. In addition, there was large variation between participants for peak power output (Figure 4a) and mean power output (Figure 4b).

**Figure 5:**
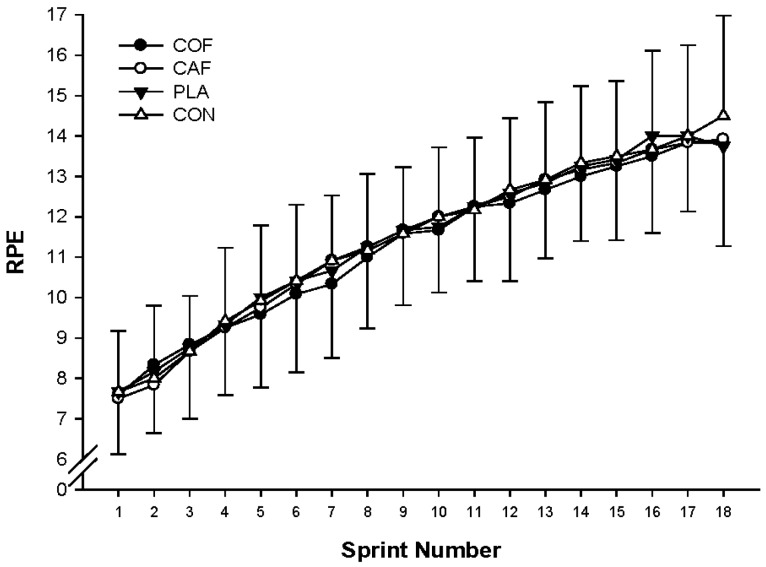
Mean RPE during repeated cycling sprints following the ingestion of caffeinated coffee, caffeine, placebo and a control condition.

**Figure 6:**
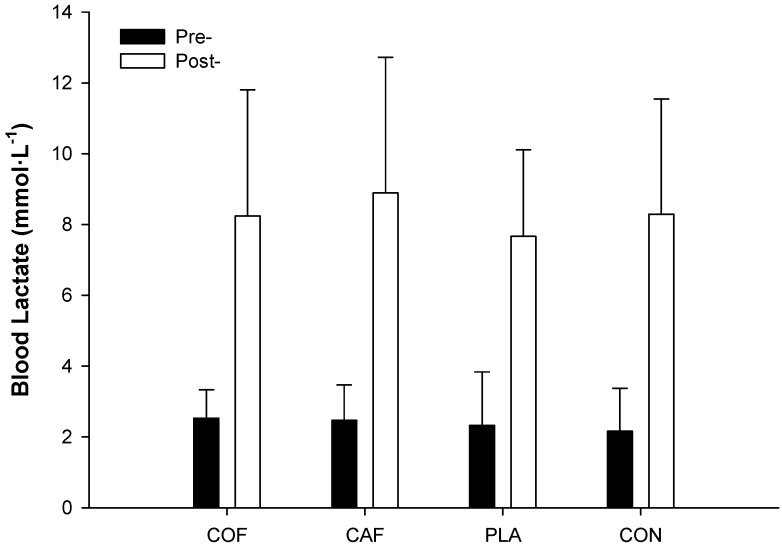
Mean blood lactate concentration before and after 18 repeated cycling sprints following the ingestion of caffeinated coffee, caffeine, placebo and a control condition.

Mean RPE was similar for all trials (Mean RPE: CAF: 11 ± 2, COF: 11 ± 2, PLA: 11 ± 2 and CON: 11 ± 2; F_3,33_ = 0.154; *p* = 0.927; $\eta_{P}^{2}$ = 0.01), although there was a moderate significant increase throughout the protocol (F_17,187_ = 3.588; *p* = 0.033; $\eta_{P}^{2}$ = 0.73; Figure 5). No significant differences in blood lactate concentration between trials were observed (F_3,33_ = 0.979; *p* = 0.415; $\eta_{P}^{2}$ = 0.08), although there was a significant main effect of time (F_1,11_ = 48.628; *p* < 0.001; $\eta_{P}^{2}$ = 0.82), with a large increase post-exercise (Figure 6).

## 4. Discussion

The aim of the present study was to determine the effect of ingesting caffeine-dose-matched anhydrous caffeine or coffee on the performance of repeated sprints on a cycle ergometer. The main findings are that neither COF nor CAF improved repeated sprint performance compared with PLA and CON. In addition, RPE and blood lactate concentration were unaffected by the ingestion of COF or CAF.

Peak power output and mean power output were not significantly different during the trials in which caffeine or coffee was consumed when compared to the placebo and control trials. In contrast, Trexler et al. [22] reported that caffeine and coffee ingestion improved sprint performance; however, a dose of 300 mg caffeine was ingested in this study and therefore participants will have consumed differing amounts of caffeine relative to their body mass. Similarly, Hodgson, Randell and Jeukendrup [21] observed that coffee and caffeine ingestion improved exercise performance by comparable magnitudes. One potential explanation for the contrasting findings is the dose of caffeine ingestion, with values ranging from ~2 to 13 mg·kg^−1^ of caffeine [28].

One of the proposed mechanisms for improved performance during exercise associated with caffeine ingestion is fatigue resistance [16]. Schneiker et al. [26] demonstrated that caffeine may cause fatigue resistance due to enriching the sodium-potassium pump within skeletal muscle. However, in the present study, peak and mean power output declined by a similar magnitude in all trials. Paton, Hopkins and Vollebregt [8] hypothesised that caffeine may actually have a negative effect in terms of fatigue on repeated exercise. This observation was also noted by Greer, McLean and Graham [34] who suggested that a dose of more than 6 mg·kg^−1^ of caffeine may be necessary to elicit an ergogenic response. Greer, McLean and Graham [34] also suggest that an initial increase in performance after caffeine ingestion may lead to fatigue developing quicker, although the finding of the present study do not support this hypothesis.

Another potential reason for the absence of an ergogenic effect following the ingestion of caffeine or coffee is training status. Crowe, Leicht and Spinks [35] suggest that the training status of participants has a substantial effect on short-term high-intensity performance. Therefore, it could be suggested that caffeine enhances performance in studies involving trained participants only, which has been observed by Schneiker et al. [26] and others [2,3,4,22]. Collomp et al. [36] suggested that specific training may be required to elicit ergogenic benefits from caffeine; improved performance in trained 100 m swimmers was observed following caffeine supplementation, but this improvement was not seen in untrained swimmers. Therefore, specific physiological adaptations to high-intensity exercise, such as enhanced regulation of the acid-base balance, may be necessary for caffeine to exert an ergogenic benefit [36]. The current study used recreationally trained participants who participated regularly in multiple sprint sports. It may therefore be possible that the variation in training backgrounds of participants in the present study contributed to the absence of an ergogenic effect of caffeine. Furthermore, this observation may also be related to the increased performance variability in untrained participants [36].

Habituation to caffeine and coffee may also have an impact on the results of the study [37]. Tarnopolsky et al. [38] demonstrated that the ergogenic effects of caffeine may not be seen in habitual caffeine users due to caffeine tolerance. It has been shown that those individuals who consume caffeine on a long term basis gain additional adenosine receptors [1]; this therefore suggests that these individuals would need to consume more caffeine for the same ergogenic effect. Two previous studies [39,40], in which participants were trained in the activity, produced contrasting results suggesting that prior habituation to caffeine is a significant factor. One suggestion for this occurrence is that Woolf, Bidwell and Carlson [39] focused only on ‘caffeine naïve’ participants, and demonstrated no ergogenic effect of caffeine during high-intensity exercise tests. However, one limitation of the present study is that habitual caffeine intake was not assessed, so this explanation is only speculative. Therefore, it would be useful for future studies to assess caffeine sensitivity.

Previous studies have indicated that caffeine generally lowers RPE [18,40], yet there were no differences in RPE observed in the present study. Furthermore, Glaister et al. [41] reported no significant differences in RPE during repeated sprints. An explanation for the lack of variation in RPE between conditions may be that during near maximal sprint efforts, differences may be not observed by participants as a ‘ceiling effect’ is created [42], therefore there is limited scope for improvement.

Although research demonstrates a positive effect during endurance activities, the results of this study reveal no effect of caffeine or coffee ingestion during repeated sprint performance in relatively untrained males. Therefore, trained athletes may be more suitable to the ergogenic effects of caffeine during high intensity exercise. In addition, considerations regarding caffeine dosage, sex, type and duration of exercise are also required.

## 5. Conclusions

Neither COF nor CAF, providing 3 mg·kg^−1^ of caffeine, improved repeated sprint performance compared with PLA and CON. In addition, RPE and blood lactate concentration were unaffected by the ingestion of COF or CAF. Therefore, anhydrous caffeine and coffee ingestion, at this dose, may not be beneficial pre-exercise nutritional strategies for repeated sprint activity in relatively untrained males.
